# Desialylation of platelets induced by Von Willebrand Factor is a novel mechanism of platelet clearance in dengue

**DOI:** 10.1371/journal.ppat.1007500

**Published:** 2019-03-08

**Authors:** Silvita Fitri Riswari, Rahajeng N. Tunjungputri, Vesla Kullaya, Fadel M. Garishah, Gloria S. R. Utari, Nur Farhanah, Gijs J. Overheul, Bachti Alisjahbana, M. Hussein Gasem, Rolf T. Urbanus, Philip. G. de Groot, Dirk J. Lefeber, Ronald P. van Rij, Andre van der Ven, Quirijn de Mast

**Affiliations:** 1 Clinical Infectious Disease Research Center, Faculty of Medicine, Universitas Padjadjaran, Bandung, Indonesia; 2 Department of Internal Medicine, Radboud University Medical Center, Nijmegen, The Netherlands; 3 Radboud Center for Infectious Diseases, Radboud University Medical Center, Nijmegen, The Netherlands; 4 Center for Tropical and Infectious Disease (CENTRID), Faculty of Medicine Diponegoro University, Dr Kariadi Hospital, Semarang, Indonesia; 5 Kilimanjaro Christian Medical Center, Kilimanjaro Clinical Research Institute, Moshi, Tanzania; 6 Department of Medical Microbiology, Radboud Institute for Molecular Life Sciences, Radboud University Medical Center, Nijmegen, The Netherlands; 7 Department of Clinical Chemistry and Haematology, University Medical Center Utrecht, Utrecht University, Utrecht, The Netherlands; 8 Department of Neurology, Translational Metabolic Laboratory, Radboud University Medical Center, Nijmegen, The Netherlands; Icahn School of Medicine at Mount Sinai, UNITED STATES

## Abstract

Thrombocytopenia and platelet dysfunction are commonly observed in patients with dengue virus (DENV) infection and may contribute to complications such as bleeding and plasma leakage. The etiology of dengue-associated thrombocytopenia is multifactorial and includes increased platelet clearance. The binding of the coagulation protein von Willebrand factor (VWF) to the platelet membrane and removal of sialic acid (desialylation) are two well-known mechanisms of platelet clearance, but whether these conditions also contribute to thrombocytopenia in dengue infection is unknown. In two observational cohort studies in Bandung and Jepara, Indonesia, we show that adult patients with dengue not only had higher plasma concentrations of plasma VWF antigen and active VWF, but that circulating platelets had also bound more VWF to their membrane. The amount of platelet-VWF binding correlated well with platelet count. Furthermore, sialic acid levels in dengue patients were significantly reduced as assessed by the binding of *Sambucus nigra* lectin (SNA) and *Maackia amurensis* lectin II (MAL-II) to platelets. Sialic acid on the platelet membrane is neuraminidase-labile, but dengue virus has no known neuraminidase activity. Indeed, no detectable activity of neuraminidase was present in plasma of dengue patients and no desialylation was found of plasma transferrin. Platelet sialylation was also not altered by *in vitro* exposure of platelets to DENV nonstructural protein 1 or cultured DENV. In contrast, induction of binding of VWF to glycoprotein 1b on platelets using the VWF-activating protein ristocetin resulted in the removal of platelet sialic acid by translocation of platelet neuraminidase to the platelet surface. The neuraminidase inhibitor oseltamivir reduced VWF-induced platelet desialylation. Our data demonstrate that excessive binding of VWF to platelets in dengue results in neuraminidase-mediated platelet desialylation and platelet clearance. Oseltamivir might be a novel treatment option for severe thrombocytopenia in dengue infection.

## Author summary

Dengue is the most common arbovirus infection in the world. A decrease in the number of blood platelets is an almost universal finding in severe dengue. Binding of the coagulation protein von Willebrand factor (VWF) and loss of sialic acid residues from the platelet membrane are two main mechanisms of clearance of senescent platelets under non-pathological conditions. Here, we show that platelets from patients with acute dengue have bound more VWF and have lost sialic acid from their membrane. Sialic acid can be cleaved by the enzyme neuraminidase. We show that neuraminidase activity in the plasma is not increased and that neither dengue virus itself nor nonstructural protein 1, a protein secreted by dengue virus, cleave sialic acid from the platelet membrane. In contrast, binding of VWF to platelets results in translocation of neuraminidase to the platelet membrane and subsequent cleavage of sialic acid. This process could be inhibited by the neuraminidase inhibitor oseltamivir, a commonly used anti-influenza drug. Altogether, our results indicate that VWF binding to platelets is increased in dengue infection, leading to the removal of sialic acid and platelet clearance. Oseltamivir may prevent this process and thus represent a novel treatment option for low platelet numbers in dengue infection.

## Introduction

Dengue is the most common arboviral infection in the world with an estimated number of 390 million annual cases, of which 96 million manifests with symptomatic disease [1]. A subset of patients with symptomatic infections develops potentially life-threatening complications in which bleeding and vascular plasma leakage are the most common [2]. To date, there is no curative therapy for dengue and clinical observation and treatment of complications remain the core principles of dengue management.

Thrombocytopenia is an early and consistent feature of dengue virus infection [3–6] and dengue complications are usually preceded by a rapid drop in platelet count [2]. Traditionally known for their key role in hemostasis, platelets are nowadays well known to have important additional functions, including regulation of inflammation and host defense [7–9] and preservation of endothelial integrity [10], especially under inflammatory conditions [11].

Circulating platelets of dengue patients are activated and excessive platelet activation may lead to platelet exhaustion [12–15], which likely contributes to thrombocytopenia and dengue complications. Other well-known mechanisms of increased platelet clearance under non-pathological conditions are the binding of von Willebrand factor (VWF) to platelets and loss of sialic acids from the platelet membrane [16]. VWF is a multimeric protein that is present in plasma, platelets, and endothelial cells. Endothelial activation results in the release of VWF from Weibel-Palade bodies into the blood circulation. Its primary function is platelet adhesion and aggregation at sites of vessel injury [17]. The antibiotic ristocetin induces binding of VWF to platelets and may cause thrombocytopenia, and as a consequence, it was removed from clinical practice [18]. We have previously shown that the plasma of children with severe dengue contains increased levels of circulating VWF in an active–platelet binding–conformation and increased VWF proteolysis [19]. Importantly, enhanced VWF binding to platelets—as seen in von Willebrand disease (VWD) type 2B - was shown to induce phagocytosis of VWF/platelet complexes by macrophages in the liver and spleen and promote thrombocytopathy by inhibition of platelet integrin α_IIb_β_3_, the platelet fibrinogen receptor [20, 21]. Increased platelet phagocytosis, as well as impaired platelet α_IIb_β_3_ function, have also been described in patients with dengue [15, 22].

Most platelet glycoproteins, including the extracellular domains of Glycoprotein 1bα (GPIbα; ‘the major VWF receptor’), are decorated with N-glycans and O-linked glycans, which are covered by sialic acids [23, 24], a family of monosaccharides with a 6-carbon backbone. Loss of terminal sialic acids, which can be mediated by human sialidase neuraminidase 1 (NEU1) or exogenous sialidase, exposes β-galactose and β-N-acetyl-D glucosamine (β-GlcNAc), which can be recognized by macrophages or the hepatic asialoglycoprotein receptor (ASGPR; also called the Ashwell–Morell receptor) [25–28]. Recently, binding of VWF to GPIbα under physiological shear was shown to induce platelet desialylation [29].

The aim of our study was to investigate these platelet clearance processes during the course of the disease in patients with dengue and their relationship with thrombocytopenia and platelet reactivity. We first show that dengue is associated with increased binding of VWF to platelets. In a follow up clinical study, we also show that circulating platelets have markedly reduced surface sialic acid. Both VWF binding and desialylation were associated with thrombocytopenia. In a set of *ex vivo* studies, we then show that VWF binding to platelets leads to NEU-1 mediated desialylation, which can be inhibited by the sialidase inhibitor oseltamivir.

## Materials and methods

### Patients and study design

Two observational clinical studies were performed in Indonesian patients with acute dengue and conducted according to the principles expressed in the Declaration of Helsinki. In study 1, the binding of VWF to circulating platelets was investigated and related to thrombocytopenia and platelet reactivity. In study 2, sialic acid on the platelet surface was determined. Both studies were exploratory in nature and therefore no sample size calculation was performed.

#### Clinical study 1

This study was conducted in Hasan Sadikin general hospital and Salamun Hospital in Bandung between January to June 2015. Febrile patients (≥37.5°C) aged ≥ 1 year old, with clinically suspected acute dengue infection, thrombocytopenia (platelet count <150 x 10^9^/l), and a positive result of either a dengue non-structural protein 1 (NS1) rapid test (PanBio Dengue Early Rapid, Alere), DENV in-house RT-PCR and/or dengue IgM (PanBio Dengue Duo cassette, Alere) were enrolled. Patients with chronic diseases, use of antiplatelet drugs or pregnancy were excluded. Medical history and physical examination were performed daily with special emphasis on bleeding manifestations and plasma leakage. Blood was drawn at admission and during the course of the infection, in which day 1–3 after onset of fever corresponds roughly with the febrile phase, day 4–6 with the critical phase, and day 7–13 with the recovery phase of a dengue infection [2, 30]. Convalescent samples were also taken >13 days after fever onset. Plasma leakage was defined as an increase in hematocrit of ≥ 20%, a single high hematocrit value (> 50% for men and > 44% for women), or by plasma leakage in the form of ascites or pleural effusion as determined by daily handheld ultrasonography, as described previously [31]. A group of healthy volunteers was recruited among hospital staff to serve as controls. None of the controls experienced a fever or other complaints in the past month and all had a normal complete blood count.

#### Clinical study 2

This study was performed from January to March 2016 in Kartini Hospital, a district hospital in Jepara, Central Java. Febrile patients clinically suspected of acute dengue infection with one of the following diagnostic criteria were enrolled: aged ≥ 5 years, positive NS1 test (PanBio Dengue Early Rapid, Alere), positive results of dengue-specific IgM or seroconversion for dengue-specific IgM or IgG (PanBio Dengue Duo cassette, Alere). Patients with chronic diseases, use of antiplatelet drugs or pregnancy were excluded. Two blood samples were drawn for this study: one sample at the critical phase or early recovery phase and one sample at the convalescence phase. Two control groups were enrolled: one group of patients with non-dengue febrile illness and a group of adult healthy volunteers recruited among hospital staff.

### Ethics statement

The first clinical study was approved by the local Medical Ethical Committee of the Medical Faculty of Padjadjaran University, Hasan Sadikin General Hospital. The second clinical study was approved by the local Medical Ethical Committee of the Faculty of Medicine, Diponegoro University, and Kartini Hospital. In both clinical studies, written informed consent was obtained before enrolment from all patients and healthy controls, and in the case of pediatric subjects, a parent or guardian provided written informed consent on behalf of the child.

### Laboratory assays clinical studies

#### Platelet reactivity and VWF binding to platelets

Venous blood was collected in 3.2% citrate anti-coagulated vacutainers (BD Biosciences). Platelet activation and reactivity were determined by flow cytometry using a method previously described [32]. Briefly, the platelet membrane expression of P-selectin (CD62P) and platelet-fibrinogen binding, which correspond with platelet degranulation and aggregation, respectively, were determined in unstimulated whole blood and after *ex vivo* stimulation with the platelet agonists adenosine-diphosphate (ADP; 7.8 μM and 31.2 μM) (Sigma-Aldrich, Zwijndrecht, The Netherlands) or PAR-1 agonist thrombin receptor-activating peptide (TRAP; 39 μM and 156.25 μM or 625 μM) (Sigma-Aldrich, Zwijndrecht, the Netherlands). VWF binding to platelets in whole blood was measured as previously described [32]. Briefly, whole blood was incubated with a mixture of HEPES-buffered saline and saturating concentrations of FITC-labeled anti-VWF (Abcam, Cambridge, UK) and PC7-labeled anti-CD61 (eBioscience), with or without the addition of ristocetin (0.433 μM, 0.577 μM and 0.77 μM) (American biochemical & pharmaceuticals). After incubation for 20 minutes at room temperature, a fixative solution (0.2% paraformaldehyde) was added. Samples were measured on a BD FACS Calibur (Bandung) or BD FACS Canto II (Jepara). Platelets were gated based on their forward- and sideward-scatter (FSC/SSC) properties and positivity for CD42b or CD61, which was defined as a median fluorescence intensity (MFI) exceeding the MFI of the matched isotype control.

#### Platelet surface sialic acid

Citrated whole blood was immediately centrifuged after blood drawing for 15 minutes at 300 g without brake to obtain platelet-rich plasma (PRP). Four million platelets were resuspended in 100 μl PBS in an Eppendorf tube and centrifuged for 8 minutes at 600 g. The platelet pellet was then stained with the platelet identification marker CD61 (anti-61 PC7, Beckman Coulter, Woerden, Netherlands) and streptavidin labelled sialic acid binding lectins *Sambucus nigra* lectin (SNA) and *Maackia amurensis* lectin II (MAL-II) (both obtained from Vector Laboratories, California, USA) for 30 minutes at room temperature (RT). Cells were then washed, fixated using 0.2% paraformaldehyde (PFA) and analyzed using flow cytometer.

#### Plasma markers

Plasma VWF antigen (VWF:Ag) and active VWF levels were determined with ELISA, as described previously [33]. Nunc maxisorb 96 wells ELISA plates were coated overnight at 4°C with 0.775 μg/ml anti-total VWF IgG (DAKO, A0082) or 5 μg/ml of anti-active VWF VHH (AU/VWF-a11). Diluted plasma samples were incubated for one hour at room temperature. Plates were washed three times and incubated with 0.55 ug/mL polyclonal rabbit anti-human VWF to capture VWF and active VWF for one hour at 37°C. Binding was detected with HRP-conjugated rabbit anti-mouse antibody. HRP activity was measured using OPD as a substrate after 30 minutes incubation at 37°C. Normal pooled plasma (NPP) was used as a standard in every assay. ADAMTS-13 activity was determined using the fluorescence resonance energy transfer (FRETS) assay for ADAMTS-13 activity (Peptides International, Inc. USA) whereby the ADAMTS-13 activity of NPP of the healthy Dutch donors was set at 100% [34]. Values obtained in the study participant samples were expressed as a percentage of NPP.

#### Plasma neuraminidase activity and transferrin glycosylation

Sialidase activity in plasma of participants in the Jepara cohort was determined with a fluorometric method with 0.45 mM 2-(4-methylumbelliferyl)α-D-N-acetylneuraminic acid as a substrate, as previously described [35]. In this assay, 30 μl plasma was mixed with equal volumes of substrate and reaction buffer of pH 7.0 and incubated at 37°C for 2 hrs. After incubation 400μl of stop solution (containing glycine and trisol) was added. The mixture was vortexed and centrifuged at 10,000 x *g* for 10 minutes. 300μl of the supernatant was transferred to a clean reaction plate and fluorescence was measured. Enzymatic activity was calculated in moles of substrate hydrolyzed per unit per time (hours). Plasma sialidase activity was also investigated by mixing platelet rich plasma (PRP) from healthy Dutch volunteers with platelet poor plasma (PPP) from participants in the Jepara cohort in a volume ratio of 1:1 for two hours at 37°C. Desialylation of platelets was analyzed by staining platelets with sialic acid binding lectins SNA and MAL-II as described above.

Transferrin glycosylation in plasma of dengue patients was analyzed using high-resolution nano liquid chromatography-chip (C8) quadrupole time of flight mass spectrometry (nanoLC-chip [C8]-QTOF MS) as previously described [35]. Briefly, plasma samples were diluted 1:10 with 0.9% NaCl solution and loaded onto a pierce spin column (Thermo Fisher) containing anti-transferrin conjugated beads. Samples were incubated for 15 minutes at room temperature, followed by 6 washing steps of 700 μl 10 mM Tris-HCl, pH 7 and a single elution (50 μl, 100 mM glycine-HCl, pH 2.7). The eluted fraction was neutralized with 1–1.5 μl 1.0 M Tris-HCl, pH 9.0 and analyzed on a microfluidic 6540 HPLC-chip-QTOF instrument (Agilent Technologies). Peak abundances were listed by Agilent BioConfirm Software and were used to calculate the ratio of sialylation by expressing the percentage of trisialotransferrin in comparison with tetrasialotransferrin (the most abundant transferrin isoform in controls).

### *In vitro* studies

#### Platelet preparation

PRP and washed platelets were isolated from 3.2% sodium-citrate-anticoagulated whole blood from healthy volunteers as previously described [36]. Briefly, whole blood was centrifuged for 15 minutes at 156x*g* without a brake to obtain PRP. PRP was further centrifuged at 330xg for 15 minutes to obtain platelets poor plasma (PPP) and platelet pellets, which were resuspended in HEPES tyrode buffer at 4x10^8^ platelets/ml.

#### Dengue NS1 and dengue virus

Washed platelets (1x10^7^ plt/ml) were exposed to different concentrations (1.25–10 μg/ml) of NS1 protein from dengue virus serotype 2 (DENV2, strain Thailand/16681/84) (Native Antigen, Oxfordshire, United Kingdom) and incubated for 4 hours at 37°C. This NS1 protein is in its native hexamer folding state and possess all post-translational modifications. It is shown to be > 95% pure and oligomeric, as demonstrated by native PAGE and Western blot analysis [37][38] and as certified by the manufacturer. In addition, washed platelets were exposed to DENV2 strain 16681 (kind gift from Claire Huang, Centers for Disease Control, Fort Collins, CO, USA). Virus stock was prepared in the *Aedes albopictus* mosquito-derived C6/36 cell line to exclude the possibility that mammalian cytokines and other inflammatory products in the virus stock would influence the results. Virus was cultured using Leibovitz’s L-15 medium (Thermo Fisher) supplemented with 10% heat-inactivated fetal calf serum (Thermo Fisher), 2% Tryptose Phosphate Broth Solution (Sigma), 1x MEM Non-Essential Amino Acids (Thermo Fisher), and 50 U/ml penicillin and 50 μg/ml streptomycin (Thermo Fisher). Briefly, cells were infected with DENV2 and after 2 hours of incubation at 28°C, the medium was replaced with fresh L-15 medium. The cells were incubated at 28°C and the supernatant was harvested 4 days post-infection, stored in aliquots at −80 °C, and titrated on BHK-15 cells by end-point dilution. Supernatant of uninfected C6/36 cells was harvested in parallel to virus stocks, for use in mock infections. Washed platelets (1x10^7^ plt/ml) were inoculated with DENV2 at a multiplicity of infection (MOI) of 3.7, or with an equal volume of mock medium, incubated for 3 or 6 hours, followed by fixation with 1% PFA to inactive DENV. Surface sialic acid was measured as described above. PFA fixation had no effect on lectin binding to sialic acid on the platelet membrane. Purified neuraminidase (NA; 200μM) from *Clostridium perfringens* (Sigma-Aldrich, Zwijndrecht, Netherlands) was used as positive control.

#### Induction of VWF-platelet binding and effect of oseltamivir

Binding of VWF to platelets was induced by exposing PRP to different concentrations of ristocetin (0–1.6 mg/ml) (American Biochemical & Pharmaceuticals, Epsom, UK) for 1hr at 37°C. VWF-platelet binding was inhibited in selected experiments by pre-incubation of PRP with 5μg/ml anti-GP1b antibodies (Abbiotec, San Diego, CA) for 30 minutes. In selected experiments, inhibition of platelet desialylation by neuraminidase inhibitors was investigated by incubation of PRP with 1–50 μM oseltamivir acid (Santa Cruz Biotechnology, Texas, USA) for 30 minutes. Plasma-derived VWF was purified from Haemate P by gel filtration chromatography as previously described [39]. The VWF was quantified using VWF antigen ELISA.

#### Flow cytometry

Surface sialic acid was measured using the lectins SNA and MAL-II as described above. In selected experiments, surface galactose expression was analyzed by incubation with streptavidin labeled *Ricinus communis* agglutinin 1 (RCA-1) lectin (vector laboratories, California, USA). Neuraminidase expression on the platelet surface was measured using antibodies against human neuraminidase-1 (anti-neu-1, Fisher scientific, Landsmeer, The Netherlands) as primary antibody and Goat-anti-rabbit IgG–Alexa Fluor 488 (Fischer scientific) as secondary antibody. Platelet VWF and expression of the lysosomal marker CD63 were measured with the following antibodies: anti-VWF FITC (Abcam, Cambridge, UK), anti-CD63 FITC, (Biolegend, San Diego, CA) and anti-CD61 PC7 (Beckman Coulter) as platelet identification marker. Platelets were incubated with saturating amounts of these antibodies for 25 minutes, fixated with 0.2% PFA for 15 minutes and measured on a Cytomics FC500 flow cytometer or a Cytoflex flow cytometer (both Beckman Coulter).

### Statistical analysis

Statistical analyses were performed using GraphPad Prism 5 software. Data are presented as geometric mean with 95% confidence intervals unless stated otherwise. The Kruskal Wallis test was used to determine significance between multiple groups. Differences between individual groups were analyzed with the Mann-Whitney test or Student’s T-test. Correlations were tested using either the Spearman correlation test (for non-normally distributed data) or Pearson correlation test (normally distributed data). A *P* value <0.05 was considered a statistically significant difference. Flow cytometry data were analyzed with Beckman Coulter Kaluza software, version 1.2. Data on transferrin glycosylation were analyzed with Agilent Mass Hunter Qualitative Analysis Software B.04.00.

## Results

### Clinical characteristics and platelet number and reactivity

In Bandung, a total number of 40 patients with acute dengue and 10 healthy adult controls were enrolled. Three patients were children aged ≤14 years old. Samples collected were divided into four groups based on the day of fever; day 1–3 (n = 20), day 4–6 day (n = 54), day 7–13 (n = 24) and day >13 days (n = 24). In Jepara, 40 patients with dengue were enrolled, together with 15 patients with non-dengue illness (acute gastroenteritis n = 9, acute respiratory tract infection n = 6) and 25 healthy controls. Ten and three of the patients with dengue or non-dengue febrile illness respectively were children. Clinical characteristics are given in Table 1. Hemorrhagic manifestations were common in both dengue cohorts (35% in Bandung and 38% in Jepara) and manifested predominantly as skin or mucosal bleeding. Ultrasonography was performed routinely in the Bandung cohort during the critical phase showing ascites, pleural effusion or a thickened gallbladder in 33 (82.5%) of the enrolled patients.

**Table 1:** Clinical characteristics cohorts Bandung and Jepara.

	Bandung		Jepara		
Variable	Dengue	Controls	Dengue	Non-dengue illness	Controls
Number	40	10	40	15	25
Male, n, (%)	27 (68)	4 (40)	21 (53)	6 (40)	14 (56)
Age, years, median (IQR)	22 (17–35)	27 (17–35)	20 (15–39)	26 (12–33)	28 (26–34)
Days of fever at presentation, median (IQR)	4 (3–5)*	NA	4.0 (2–6)	4.1 (2–6)	NA
NS1 positive, n (%)	38 (95)	NA	16 (40)	0 (0)	NA
Bleeding manifestations, n (%)	14 (35)	NA	15 (38)	0 (0)	NA
Bleeding type, n (%)		NA			NA
Petechia	3 (7.5)		7 (17.5)		
Gum bleeding	8 (20.0)		5 (12.5)		
Epistaxis	5 (12.5)		6 (15)		
Melena	2 (5.0)		3 (7.5)		
Hypermenorrhea	1 (2.5)		0 (0)		
Classification, n (%)^#^					
DF	8 (20)	NA	25 (62)	NA	NA
DHF	32 (80)	NA	15 (38)	NA	NA
Full blood count at presentation, median (IQR)*					
Hemoglobin, gr/dL	14.2 (13.5–15.2)	13.4 (12.7–16.1)	13.8 (12.4–14.1)	13.6 (12.8–13.9)	13.9 (12.9–14.2)
Platelet count, 10^9^/L	62 (34–101)	265 (237–312)	46 (29–72)	152 (104–211)	261 (197–309)
Hematocrit, %	42.0 (40.0–44.8)	41.0 (38.3–46.3)	41.7 (38.1–41.8)	42.2 (37.7–42.8)	NA

^#^ DF, dengue fever; DHF, dengue hemorrhagic fever (defined as dengue with hemorrhagic manifestations, plasma leakage, and platelet count <100x10^9^/L)

NA, not applicable

**Fig 1:**
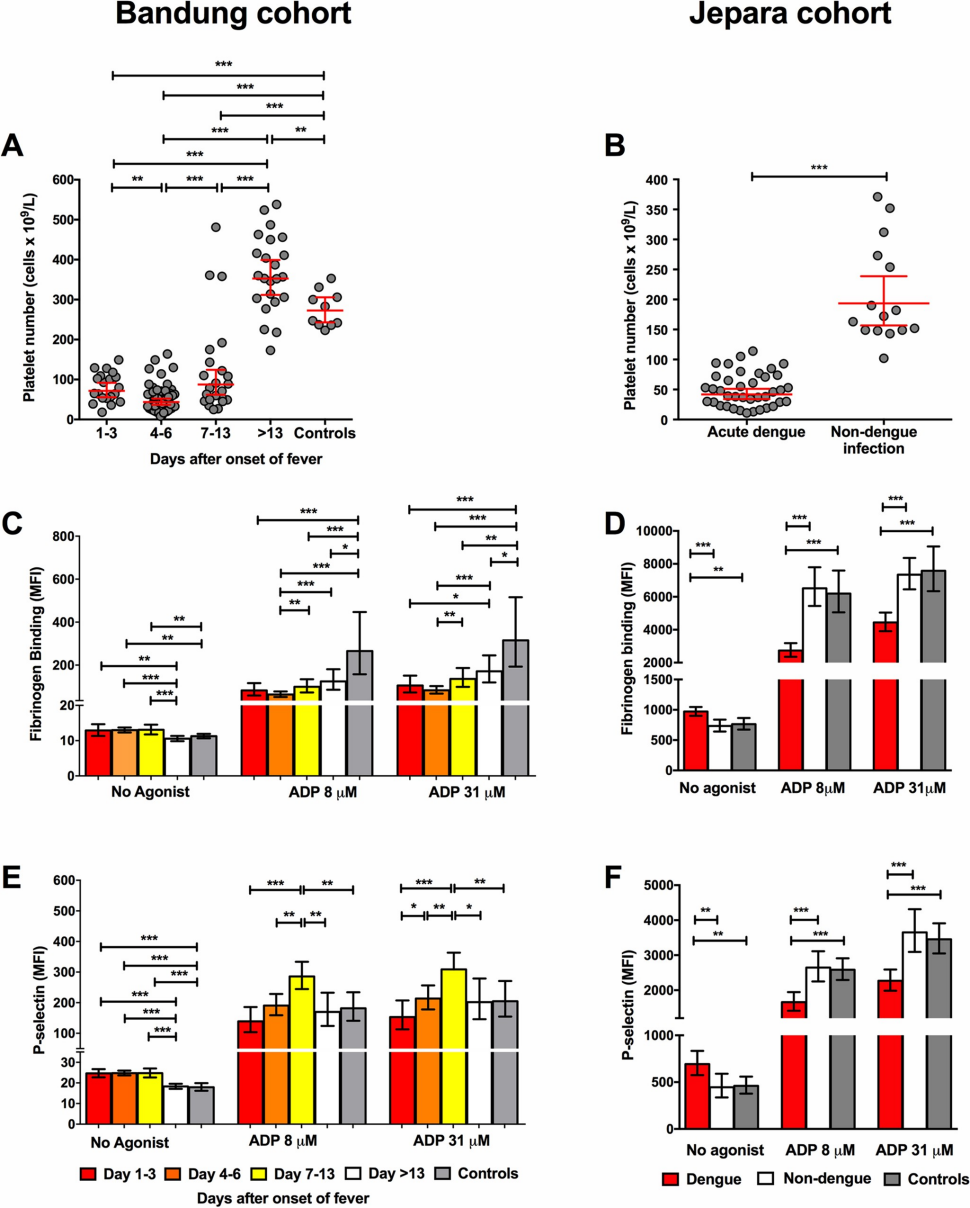
Platelet number and platelet activation with impaired reactivity in patients with dengue infection. **(A, B)** Platelet number at enrolment in cohorts from Bandung and Jepara. Binding of fibrinogen **(C, D)** to platelets and platelet P-selectin expression **(E, F)** in unstimulated samples and after *ex vivo* stimulation with two concentrations of ADP. Platelet P-selectin expression and binding of fibrinogen were measured using flow cytometry and are expressed as median fluorescence intensity (MFI) in arbitrary units. Data depicted as geometric mean with 95% confidence interval. Differences between groups were analyzed using the Mann-Whitney U test, *P < 0.05, ** *P*<0.01, ***P<0.001.

Fig 1A and 1B shows platelet numbers in the Bandung and Jepara cohorts. Platelets numbers in healthy controls were not determined. As expected, platelet numbers were lowest on day 4–6 after onset of fever, which roughly corresponds with the critical phase of dengue. Fig 1C–1F shows data of platelet activation and reactivity to *ex vivo* stimulation with the platelet agonist ADP. Compared with healthy controls, circulating platelets of dengue patients were activated on day 1–3 and day 4–6 after fever onset, as shown by increased expression of P-selectin and fibrinogen binding to integrin α_IIb_β_3_. In conjunction with this, *ex vivo* reactivity to stimulation with ADP was significantly reduced on these days compared with later phases and healthy controls, indicating platelet dysfunction. Similar findings were found for platelet reactivity towards the platelet agonist TRAP (S1 Fig). These findings confirm our earlier findings that platelets in patients with dengue are activated, but also functionally impaired [15].

### Increased VWF binding to platelets

**Fig 2:**
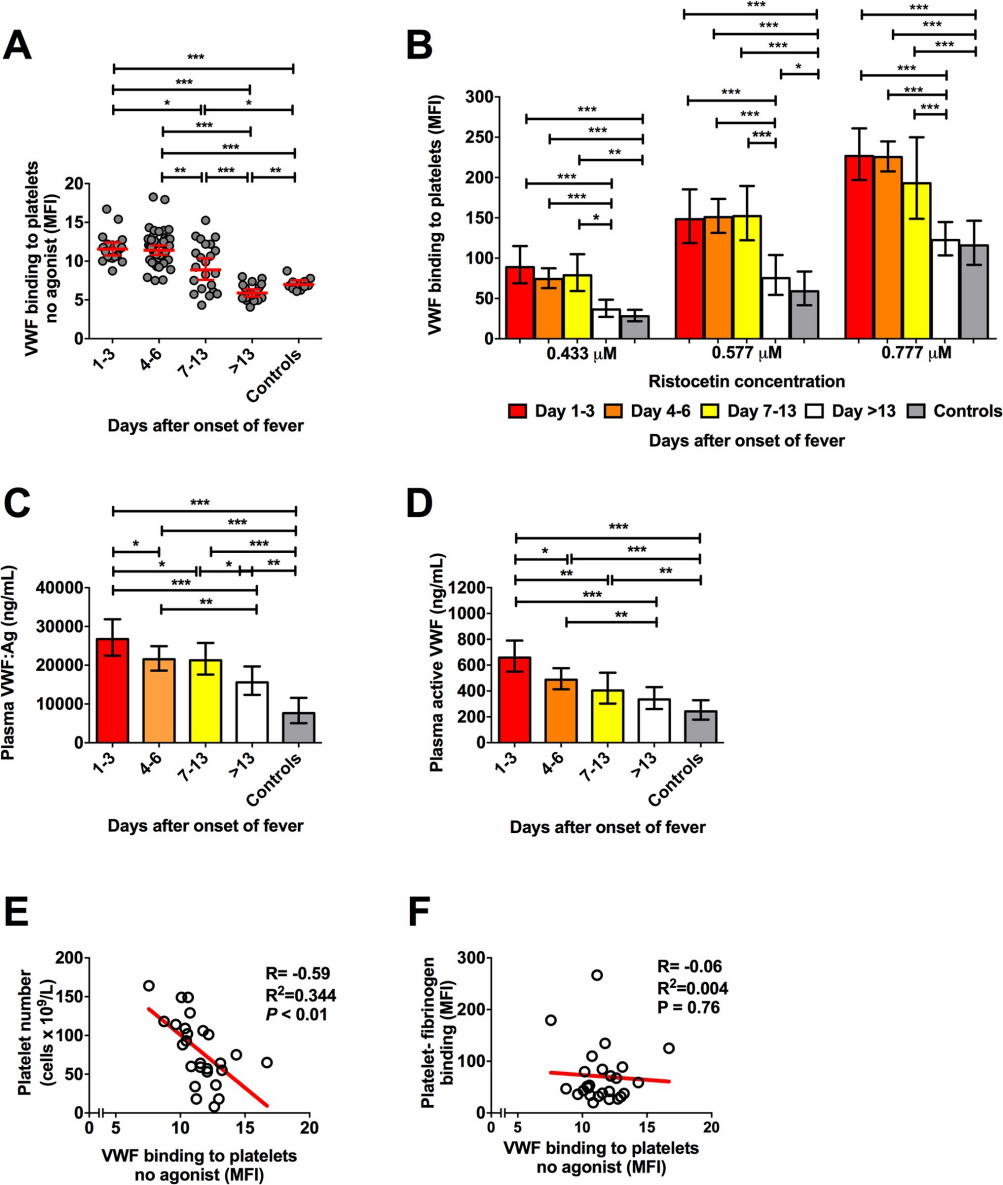
Increased VWF-platelet binding and plasma VWF:Ag and active VWF levels in dengue patients. **(A)** Platelet-VWF binding measured by flow cytometry in unstimulated samples (line is geometric mean with 95% confidence interval) and **(B)** after *ex vivo* stimulation with increasing concentrations of ristocetin in participants with acute dengue and healthy volunteers. **(C)** Time course of plasma VWF:Ag levels and **(D)** active VWF levels in the same participants. **(E)** Pearson correlation between platelet-VWF binding and platelet number in enrolment samples and **(F)** fibrinogen binding after *ex vivo* stimulation with high dose (156μM) TRAP in enrolment samples. Data shown are individual values or aggregated data as geometric mean with 95% confidence interval. Differences between groups were analyzed using the Mann-Whitney U test, *P < 0.05, ** *P*<0.01, ***P<0.001.

Plasma levels of active VWF levels are increased in patients with dengue [19], but it is unknown whether this results in higher binding of VWF to platelets. We, therefore, determined the binding of VWF to platelets in the Bandung cohort using flow cytometry. We observed a significantly (*P*<0.001) higher binding of VWF to circulating platelets of patients in the acute or critical phase of dengue compared to healthy controls (Fig 2A; gating strategy shown in S2 Fig). When plasma VWF was further activated by *ex vivo* incubation of whole blood with different concentrations of ristocetin, VWF-platelet binding increased. Higher values were obtained in the febrile and critical phase than in the convalescence phase and in healthy controls (Fig 2B). Median plasma VWF antigen (VWF:Ag) concentrations were approximately three fold higher in patients in the acute phase than in controls (median, (IQR); 27.9 μg/ml (21.6–38.8 μg/ml) vs. 8.4 μg/ml (4.4–14.1 μg/ml); *P*< 0.01) and remained elevated throughout the convalescent phase (Fig 2C). Plasma concentrations of active VWF, which means VWF in which the platelet binding epitope on the A1 domain is exposed, were two-fold higher in the acute phase than in controls (median (IQR) 629 ng/ml (461–860 ng/ml) vs. 300 ng/ml (151–319 ng/ml); *P*<0.01) (Fig 2D). Levels of ADAMTS-13, an enzyme that cleaves prothrombotic ultra-large VWF, was significantly lower in patients around the critical phase (day 4–6 after fever onset) compared with the convalescent phase (day >13) and healthy controls (S3 Fig).

Platelet-VWF binding was inversely correlated with platelet numbers in enrolment samples (Pearson R = -0.59; *P*< 0.001) (Fig 2E). Increased VWF-platelet GP1b interaction was reported to be associated with inhibition of platelet α_IIb_β_3_ [21], but we found no correlation between platelet-VWF binding and TRAP- or ADP-induced platelet fibrinogen binding (Fig 2F, data high dose TRAP shown). There was also no correlation between platelet-VWF binding and plasma VWF levels (R = -0.09; *P* = 0.68), active VWF levels (R = 0.08; p = 0.69) or ADAMTS-13 levels (R = -0.19; *P* = 0.39) (S3 Fig). These results show for the first time that VWF binding to platelets is a feature of dengue which may contribute to dengue-associated thrombocytopenia.

Finally, we compared platelet parameters between patients with and without bleeding, as well as those with and without plasma leakage. No significant differences in platelet count, platelet-VWF binding, plasma VWF and active VWF levels were found between subjects with or without bleeding complications and between those with or without plasma leakage, except for a significantly higher plasma VWF in the in the plasma leakage group (S4 Fig).

### Platelet desialylation in dengue infection

**Fig 3:**
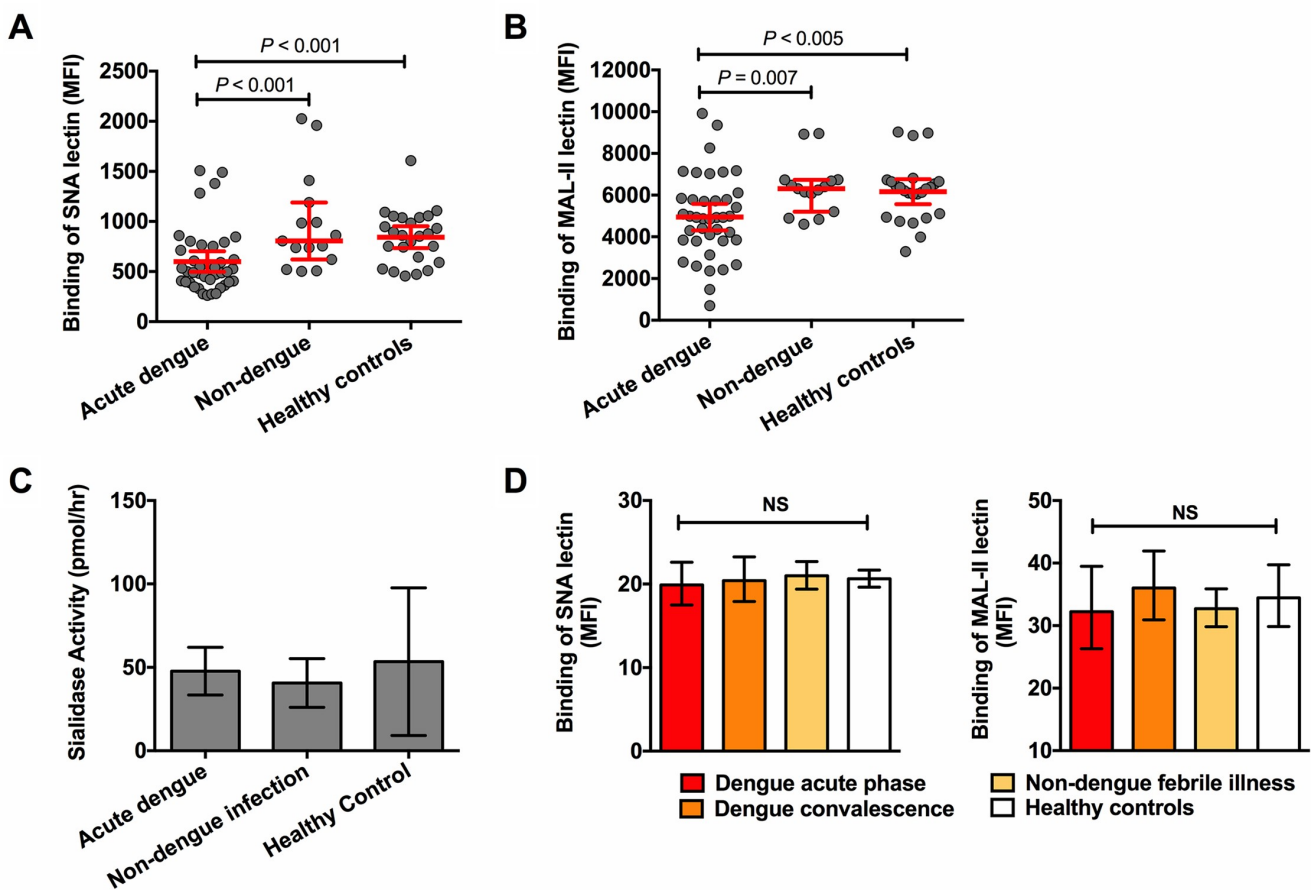
Platelet desialylation in acute dengue and plasma sialidase activity. Binding of the lectins **(A)** SNA and **(B)** MAL-II to platelet sialic acid residues measured by flow cytometry, in patients with acute dengue (n = 40), non-dengue febrile illness (n = 15) and healthy controls (n = 20) in Jepara. **(C)** Plasma sialidase activity measured as moles of substrate (2-(4-methylumbelliferyl)α-D-*N*-acetylneuraminic acid) hydrolyzed per hour. **(D)** Binding of SNA and MAL-II lectins to platelets following incubation of platelet rich plasma from healthy Dutch volunteers with platelet poor plasma from patients with dengue (acute and convalescence sample), non-dengue febrile illness and healthy controls (n = 6 per group). Data expressed as geometric mean with 95% CI. Samples depicted in panels A-B were analyzed on a BD FACS Calibur in Jepara, and samples in panel D on a Beckman coulter FC500 flow cytometry in The Netherlands, which explains the differences in MFI values. Differences between groups were analyzed using the Mann-Whitney U test, *P < 0.05, ** *P*<0.01, ***P<0.001. NS depicts statistically non-significant result across groups using Kruskal-Wallis test.

Next, in the Jepara cohort, we determined whether there was also loss of sialic acid from the platelet membrane in dengue infection. Platelet surface sialic acid was measured in patients in the critical or early recovery phase of dengue (median 7 days after fever onset) using the lectins SNA and MAL-II, which primarily binds α-2,6-sialoglycans and MAL-II α-2,3-sialoglycans, respectively. The median (IQR) platelet count at the time of measurement was 57 (37–81) x10^9^/L. Binding of both lectins to sialic acid residues was significantly reduced in patients with dengue, compared with patients with non-dengue febrile illness and healthy controls (Fig 3A and 3B; gating strategy shown in S5 Fig). In 12 participants, SNA and MAL-II binding was also measured in the convalescence phase when platelets numbers had normalized. Both platelet SNA and MAL-II had increased significantly compared with the early dengue phase with median MFI values for SNA of 762 (663–857) vs. 537 (439–752; *P* = 0.003; Wilcoxon signed rank test) and for MAL-II of 6246 (5171–7188) vs. 5096 (4435–6004; *P* = 0.001). In the early dengue phase, platelet count correlated significantly with SNA binding (Spearman R 0.48; *P* = 0.002), but not with MAL-II binding (R 0.28; *P* = 0.08). In addition, there was also no correlation between SNA or MAL-II binding and levels of VWF:Ag (SNA R = -0.2; *P* = 0.25 and MAL-II R = 0.13; *P* = 0.46) or of active VWF (SNA R = -0.2; *P* = 0.25 and MAL-II R = 0.13; *P* = 0.46). We compared dengue patients with and without bleeding in the Jepara cohort and did not find statistically significant differences in platelet sialic acid expression or platelet reactivity (S6 Fig).

Next, we investigated mechanisms underlying desialylation. First, we tested whether plasma sialidase activity was increased in acute dengue with a functional sialidase activity assay. As shown in Fig 3C, sialidase activity in the plasma of acute dengue patients was not increased in comparison to the other groups. Further proof that platelet desialylation in dengue infection does not result from increased plasma sialidase activity was provided by mixing PRP from healthy Dutch volunteers with PPP (1:1 volume ratio) from randomly selected patients with dengue (sample from acute and convalescent phase), non-dengue febrile illness or healthy controls (n = 6 each group). As shown in Fig 3D, no differences in platelet desialylation across the groups were observed. Finally, to further confirm these results, we performed glycoprofiling of intact plasma transferrin using mass spectrometry in five dengue patients with the highest plasma sialidase activity in the functional assay [35]. Loss of sialic acid was calculated using the ratio of the undersialylated trisialo-glycosylated transferrin form and this ratio was below the upper reference of normal (<5%) in all five patients (Table 2).

**Table 2:** Sialidase activity and plasma transferrin glycosylation of patients with dengue.

	Sialidase activity (pmol/hr)	Sialic acid loss (%)
Dengue patient 1	99	2.8
Dengue patient 2	101	2.6
Dengue patient 3	103	1.9
Dengue patient 4	106	3.6
Dengue patient 5	123	2.8

Sialic acid loss upper reference of normal: <5%

### *In vitro* studies

**Fig 4:**
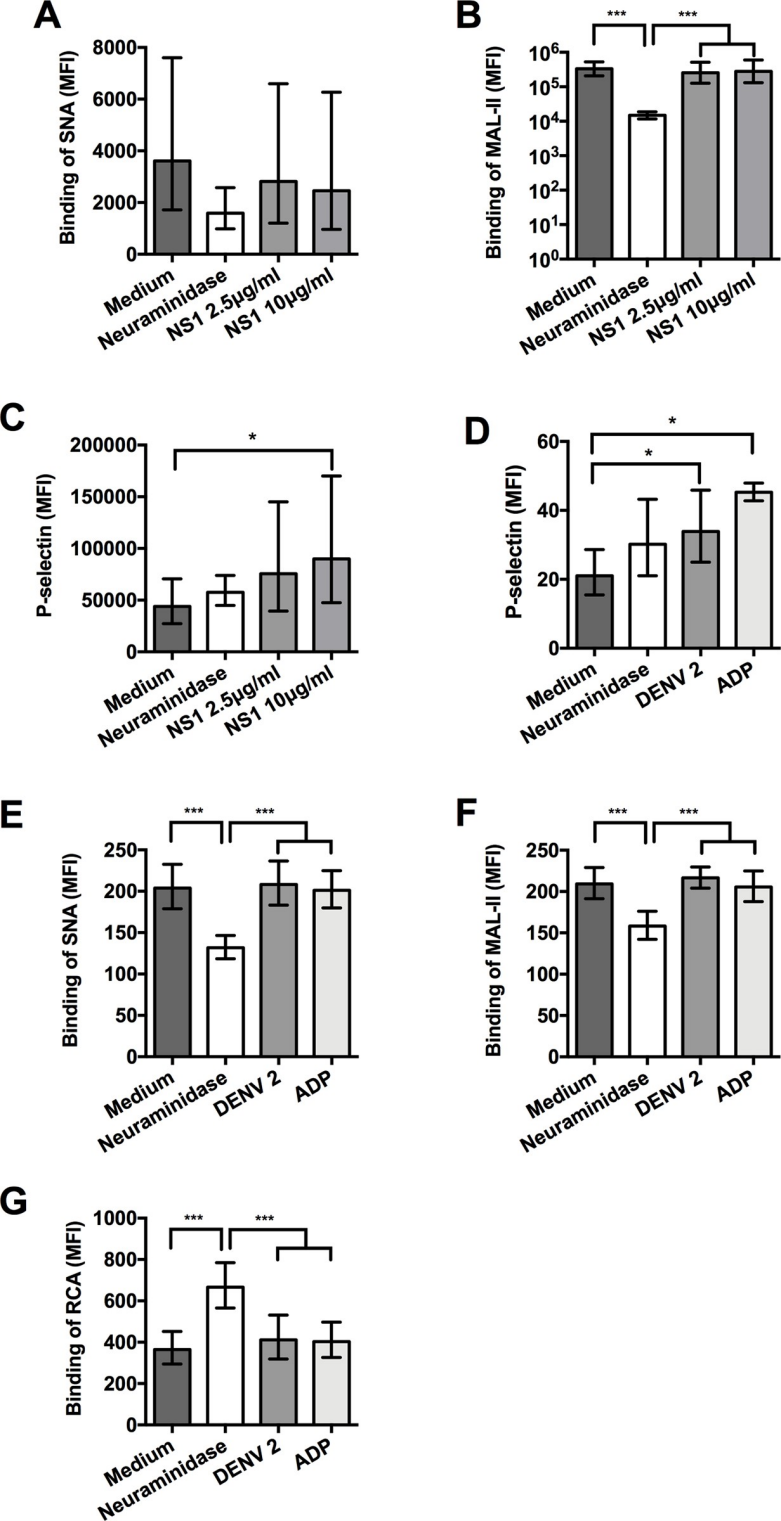
Dengue non-structural protein 1 (NS1) nor dengue virus (DENV) induce platelet desialylation. Binding of **(A)** SNA or **(B)** MAL-II lectins to washed platelets or **(C)** the expression of P-selectin after incubation with two concentrations of DENV2 NS1 protein for 4 hrs at 37°C (n = 7 platelet donors). **(D)** shows expression of platelet P-selectin and **(E-G)** the binding of SNA, MAL-II, or RCA lectins to washed platelets following incubation with dengue virus type 2 (DENV 2) for 3 hrs at 37°C (n = 8 platelet donors). Purified neuraminidase from *C*. *perfringens* (100 mU) and the platelet agonist adenosine diphosphate (ADP) at 125 μM were used as positive controls. Mock infection with supernatant of uninfected C6/36 cells harvested at the same time as DENV2 stocks was used as negative control. Data are shown as geometric mean with 95% confidence interval. Samples depicted in panels A-C were analyzed on a Beckman coulter Cytoflex, and samples in panel D-G on a FC500 flow cytometer, which explains the differences in MFI values. Differences between groups were analyzed using the Mann-Whitney U test, *P < 0.05, ** *P*<0.01, ***P<0.001.

Other possible mechanisms responsible for removal of sialic acid from platelets in dengue infection were further explored in a series of *in vitro* experiments. A recent study showed that dengue virus NS1 protein is able to induce the expression of sialidase and heparanase in endothelial cells, leading to desialylation of the endothelial glycocalyx layer [38]. We, therefore, investigated whether NS1 also induces desialylation of platelets. Incubation of washed platelets or PRP with different concentrations of NS1 up to four hours did, however, not reduce binding of SNA or MAL-II lectins to sialic acid residues, nor did it increase the binding of RCA to galactose residues or surface expression of Neu1 on platelets indicating that NS1 did not induce platelet desialylation (Figs 4A, 4B and S7A). As expected, desialylation was observed using purified neuraminidase from *C*. *perfringens* as a control. NS1 did induce platelet activation, as indicated by increased P-selectin expression (Fig 4C). Next, we investigated whether DENV itself would induce removal of sialic acid. DENV is known to activate platelets [12], which we confirmed in our experiments by finding increased platelet P-selectin expression (Fig 4D). However, removal of sialic acid, as evidenced by a reduction in the binding of SNA or MAL-II, or increased binding of RCA lectin, which is a lectin that binds to desialylated β-galactose residues, was not observed following incubation of washed platelets with DENV2 for 3 or 6 hours (Fig 4E, 4F and 4G; only data at 3 hours are shown).

**Fig 5:**
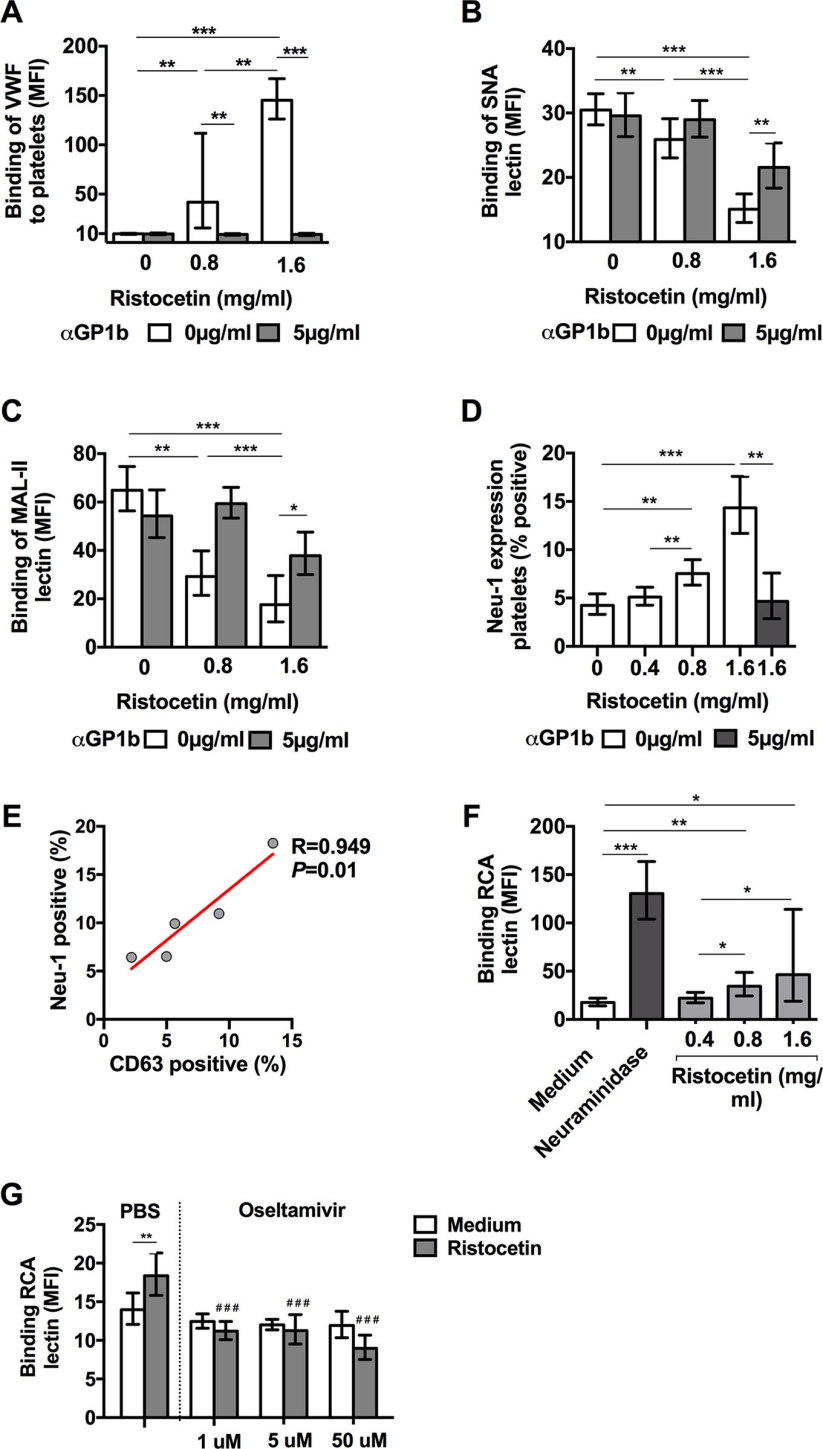
Binding of VWF to platelets induces platelet desialylation which can be inhibited by oseltamivir. Binding of **(A)** von Willebrand factor (VWF), **(B)** SNA and **(C)** MAL-II lectins to platelets and **(D)** platelet membrane expression of Neuraminidase-1 (Neu-1) after exposing platelet rich plasma (PRP) of healthy volunteers to ristocetin for 1 hr at 37°C in presence and absence of GPIb receptor blocking antibodies. **(E)** Pearson correlation between the expression of Neu-1 and the lysosomal marker CD63 on the platelet membrane after incubation PRP with ristocetin (1.6mg/ml for 1 hour). **(F)** Binding of RCA lectin to galactose or N-acetylgalactosamine residues on platelets after exposing PRP to ristocetin **(G)** with and without the addition of increasing concentration of oseltamivir. Purified neuraminidase from *C*. *perfringens* (100mU) was used as a positive control. Data were analyzed using Student’s T-tests and presented as geometric mean with 95% confidence interval. *** *P* < 0.001, ** *P* < 0.005, * *P* < 0.05; ^###^ *P* < 0.001 when samples were compared with ristocetin-treated samples that were unexposed to oseltamivir. Data were from on 5–7 platelet donors in ≥ 2 independent experiments.

Next, because our clinical data showed that platelet-VWF binding is increased in dengue infection, we explored whether VWF binding to platelets could be responsible for desialylation of platelets. To induce VWF binding to platelets, we exposed PRP of healthy volunteers to increasing concentrations of ristocetin, which indeed resulted in a dose-dependent increase in VWF-platelet binding (Fig 5A). In the same time, surface sialic acids were reduced as evidenced by a reduction in binding of the lectins SNA and MAL-II. These effects were dependent on VWF binding to GPIbα as blocking the VWF with anti-GPIbα antibodies prevented desialylation (Fig 5B and 5C). VWF-mediated platelet desialylation was confirmed using washed platelets and purified VWF whereby addition of ristocetin resulted into exposure of galactose residues (detected by RCA lectin binding) in a dose response manner (S7B Fig). We further show that ristocetin-induced VWF-platelet binding increases the expression of Neu1 on the platelet surface (Fig 5D). Neu1 is stored in platelet lysosomes and the Neu1 expression on the membrane correlated strongly (R 0.95; *P* = 0.01) with the lysosomal marker CD63 (Fig 5E). Previous studies showed that the neuraminidase inhibitor oseltamivir is able to inhibit Neu1 activity in platelets [40]. We confirmed these findings by showing that ristocetin induces the binding or RCA lectin to platelets (Fig 5F), which can be inhibited by oseltamivir acid in a concentration as low as 1μM, which is equivalent to plasma levels of the drug [41] (Fig 5G). Lower concentrations of oseltamivir resulted into inconsistency in the RCA lectin binding.

## Discussion

In this study, we describe a new mechanism of platelet clearance and thrombocytopenia in dengue infection. The release of VWF by activated endothelial cells results in excessive VWF binding to platelets, leading to loss of surface sialic acid. We show for the first time that a dengue infection induces a marked increase in VWF binding to circulating platelets, which was inversely related to platelet number. We further show for the first time that circulating platelets in dengue patients have lost surface sialic acid. This occurred in the absence of increased plasma sialidase activity. We further investigated the mechanisms underlying platelet desialylation in dengue infection *in vitro* and identified increased VWF binding to platelets as the most likely mechanism. Platelets are important in preservation of endothelial integrity [10]. We speculate that the course of events in dengue infection is that endothelial cell activation in the setting of dengue infection leads to release and activation of VWF. The resulting VWF-mediated platelet clearance may in turn contribute to the vascular leakage syndrome that is characteristic of severe dengue.

We and other groups have previously shown that patients with acute dengue have higher VWF plasma levels [19, 42]. Under normal circumstances, VWF does not bind to GPIbα on platelets, but does so only after a conformational change exposing its active A1 domain. Active VWF can be measured using a specific nanobody, and here, we confirm our earlier findings that the amount of active VWF in the circulation is markedly increased in dengue infection [19]. Our present study adds to these earlier data that we now also show directly, using a novel flow cytometry assay, that more VWF has bound to circulating platelets. It thereby supports the notion that VWF is important in the etiology of dengue-induced thrombocytopenia, as binding of VWF to platelets is expected to result in platelet clearance. It is also in concordance with our earlier observations that VWF:Ag is being consumed in children with severe dengue [19]. In contrast to our current findings, VWF:Ag levels in these children admitted to the ICU with severe dengue showed an increasing trend towards discharge, while levels of VWF:propeptide decreased. Because VWF:propeptide is secreted in equimolar amounts as VWF:Ag, this time course was suggestive for VWF consumption. In the current study, VWF consumption was probably less pronounced as most participants had dengue disease without severe complications.

Another important finding of our study was that circulating platelets in dengue patients contained less surface sialic acid. Removal of sialic acid exposes terminal β-galactose and β-GlcNAc resulting in accelerated platelet clearance via Ashwell–Morell receptor [25–28]. Removal of 8–10% of platelet sialic acid was reported to be sufficient to cause platelet clearance from the circulation [43]. Recently, VWF binding to platelets by botrocetin or by platelet refrigeration was shown to induce unfolding of the mechanosensory domain of the GPIbα subunit, leading to GPIb-IX–mediated signaling in the platelet, including calcium mobilization, phosphatidylserine exposure, and likely trafficking of Neu1 to the platelet surface and subsequent desialylation of platelet glycoproteins [29],[44]. We confirm these findings *in vitro* by demonstrating that induction of VWF binding to platelets with ristocetin results in increased expression of Neu1 on the platelet surface and platelet desialylation. We cannot exclude that ristocetin may execute a VWF-independent effect on platelet desialylation, as previous studies suggested that ristocetin has direct effects on GPIbα and other platelet receptors [45, 46]. However, our findings that ristocetin did not induce platelet desialylation in the presence of GPIbα blocking antibodies or in washed platelets in absence of VWF supports the conclusion that the effects of ristocetin on platelet desialylation are through induction of VWF binding to platelet GP1bα. In addition, the snake venom protein botrocetin, which induces VWF binding to platelets but does not bind GPIbα, also triggers platelet desialylation [44].

Our present study also supports findings from previous studies that platelets are activated during a DENV infection [12, 15]. Despite our observation that activating platelets *ex vivo* using ADP did not result in removal of sialic acid from the platelet membrane, we cannot exclude that *in vivo* platelet activation results in trafficking of Neu1 to the membrane where it may desialylate GP1b, leading to increased binding of VWF to the platelet.

We also investigated other possible mechanisms of platelet desialylation in dengue infection. NS1 was recently shown to induce sialidase in endothelial cells, leading to degradation of endothelial bound sialic acid [47], but we did not find platelet desialylation upon exposure of platelets to NS1. In addition, dengue virus does not express neuraminidase, in contrast to other viruses as influenza or bacteria as *Streptococcus pneumoniae*. Incubation of platelets with cultured dengue virus did induce platelet activation, as previously reported [12–14], but did not induce platelet desialylation. This was further supported by the fact that sialidase activity in plasma of dengue patients was not increased, that plasma of dengue patients failed to induce desialylation of platelets of healthy volunteers and that plasma proteins (transferrin) of dengue patients were not desialylated. The latter is in contrast to an earlier study by Rajendiran *et al*. [48], who reported increased desialylation of plasma proteins in patients with dengue.

*In vitro*, VWF-induced platelet desialylation could be circumvented by the neuraminidase inhibitor oseltamivir acid, which is widely used worldwide in the treatment of influenza infections. Interestingly, over the past years, different authors have reported that oseltamivir may increase platelet counts in conditions such as immune thrombocytopenic purpura [49, 50], sepsis [51] and suspected influenza [52]. We are currently carrying out a phase 2 randomized clinical trial to study the effect of oseltamivir on thrombocytopenia and plasma leakage in dengue infection (ISRCTN35227717). In addition, antagonists of VWF, such as the anti-VWF aptamer ARC1779 are nowadays available. ARC1779 was able to reverse thrombocytopenia in VWD type 2B, a condition characterized by excessive binding of VWF to platelets [53]. This reinforces the importance of VWF in platelet clearance and supports the possible use of VWF antagonists as adjunctive therapy for dengue-associated thrombocytopenia.

Our present study confirms our earlier finding that acute dengue infections are not only associated with thrombocytopenia but also platelet dysfunction with hyporeactivity to *ex vivo* activation [15]. This platelet dysfunction, which may contribute to bleeding complications, may be a consequence of profound platelet activation in the circulation resulting in secondary loss of function. Additional proof for the occurrence of platelet exhaustion in dengue infection was recently provided by a quantitative proteomics study of platelet contents showing exhaustion of the granule-stored PF4/CXCL4 [54]. Gain of function mutations in VWF, as seen in von Willebrand disease type 2B were shown to impair activation of the α_IIb_β_3_ integrin. However, this mechanism appears to be less important in dengue infection, as there was no inverse correlation of platelet-VWF binding with platelet-fibrinogen binding in our cohort.

Different limitations of our studies should also be acknowledged. First, VWF binding to platelets and platelet sialic acid were measured in different cohorts, preventing us to analyze a direct correlation between both parameters. We were able to correlate platelet SNA and MALII lectin binding with plasma levels of active VWF, but this did not show a significant correlation. In our opinion, however, this does not disprove the hypothesis that excessive platelet-VWF binding is responsible for platelet desialylation in dengue infection, because VWF binding to platelets did not correlate with active VWF plasma levels in our cohort. Second, it was not possible to measure the expression of Neu1 on the platelet membrane in the patient samples. Third, the proposed mechanisms for platelet desialylation were analyzed using *in vitro* studies and samples from healthy individuals, rather than dengue-infected patients due to limited availability of patient material.

In conclusion, acute dengue infections induce the binding of VWF to platelets, which results in the removal of sialic acids from the platelet surface by the actions of endogenous neuraminidase. Neuraminidase inhibitors such as oseltamivir might represent a novel therapeutic option for dengue-associated thrombocytopenia.

## Supporting information

S1 Fig Platelet activation with impaired reactivity to TRAP in patients with acute dengue. Binding of fibrinogen to platelets and platelet P-selectin expression in unstimulated samples and after *ex vivo* stimulation with two concentrations of TRAP. **(A, B)** Data from Bandung cohort with longitudinal data from the different days of fever in dengue patients and in healthy controls. Platelet P-selectin expression and binding of fibrinogen were measured using flow cytometry and are expressed as median fluorescence intensity (MFI) in arbitrary units. Data depicted as geometric mean with 95% confidence interval. Differences between groups were analyzed using the Mann-Whitney U test, *P < 0.05, ** *P*<0.01, ***P<0.001.
(DOCX)

S2 Fig Flow cytometry gating strategy for determination of VWF binding to platelets. Platelets were gated based on forward and side scatter characteristics **(A)**, followed by positivity for the platelet marker CD61-PC7 **(B)**. The Median fluorescence intensity (MFI) of anti-VWF after stimulation **(C)** without agonist and **(D)** after *ex vivo* VWF-activation with ristocetin (0.777 μM). **(E)** Observed differences in VWF binding to platelet with marker Anti-VWF-FITC in unstimulated sample and after *ex vivo* stimulation of 0.777 μM.
(DOCX)

S3 Fig ADAMTS-13 activity in Bandung cohort. (**A**) Data from different days of fever in dengue patients and in healthy controls. Data are shown as geometric mean with 95% confidence interval. Differences between groups were analyzed using the Mann-Whitney U test. (**B-D**) The correlation between VWF binding to platelets without any agonist stimulation and plasma VWF, VWF activation factor and ADAMTS13 activity is shown. Analysis were done using Pearson correlation coefficient. *P < 0.05, ** *P*<0.01, ***P<0.001, **** *P*<0.0001.
(DOCX)

S4 Fig Differences in platelets and VWF parameters between dengue patients with and without bleeding, and patients with and without plasma leakage. Data shown are platelet numbers (**A** and **B**), VWF binding to platelets in the absence of an agonist (MFI) (**C** and **D**), Plasma VWF:Ag levels (**E** and **F**) and plasma active VWF levels (**G** and **H**). Differences between groups were analyzed using the Mann-Whitney U test, *P < 0.05, ** *P*<0.01, ***P<0.001.
(DOCX)

S5 Fig Flow cytometry gating strategy for determination of sialic acid expression on platelets. Platelets were gated in P0 based on forward and side scatter characteristics (**A**) followed by positivity of the platelet marker CD61-PC7 in P1 (**B**). The median fluorescence intensity (MFI) of PE-labeled SNA lectin on platelets is determined from gate P1 (**C**, higher expression, and **D**, lower expression of sialic acid).
(DOCX)

S6 Fig Sialic acid expression and platelet reactivity in dengue patients with or without bleeding. Binding of the lectins (**A**) SNA and (**B**) MAL-II to platelet sialic acid residues were measured by flow cytometry in dengue patients with (n = 22) and without bleeding (n = 18). Platelet P-selectin expression and binding of fibrinogen to platelets in unstimulated samples and after *ex vivo* stimulation with two concentrations of ADP (**C**, **D**). Platelet P-selectin expression and binding of fibrinogen were measured using flow cytometry and are expressed as median fluorescence intensity (MFI) in arbitrary units. Data are expressed as geometric mean with 95% CI. Differences between groups were analyzed using the Mann-Whitney U test, *P < 0.05, ** *P*<0.01, ***P<0.001.
(DOCX)

S7 Fig Platelet desialylation is mediated by VWF binding to platelets. **(A)** Expression of Neuraminidase 1 (Neu-1) and binding of RCA lectin and VWF to platelets after incubation with two concentrations of DENV NS1 protein for 4 hrs at 37°C (n = 7 platelet donors). **(B)** Binding of VWF and RCA to platelets after incubating washed platelets with increasing concentrations of purified VWF and 1.6mg/ml of ristocetin for 1 hr at 37°C (n = 5 platelet donors). Purified neuraminidase from *C*. *perfringens* (100 mU) was used as positive control. Samples were analyzed using Beckman coulter Cytoflex flow cytometry. Data are shown as geometric mean with 95% confidence interval. Differences between groups were analyzed using the Mann-Whitney U test, *P < 0.05, ** *P*<0.01, ***P<0.001.
(DOCX)
