# Supplementary material for: Desialylation of platelets induced by Von Willebrand Factor is a novel mechanism of platelet clearance in dengue
Source: PLoS Pathog. 2019 Mar 8;15(3):e1007500. doi: 10.1371/journal.ppat.1007500 (PMC6426266; doi:10.1371/journal.ppat.1007500)
Supplement: S1 Fig — Binding of fibrinogen to platelets and platelet P-selectin expression in unstimulated samples and after ex vivo stimulation with two concentrations of TRAP. (A, B) Data from Bandung cohort with longitudinal data from the different days of fever in dengue patients and in healthy controls. Platelet P-selectin expression and binding of fibrinogen were measured using flow cytometry and are expressed as median fluorescence intensity (MFI) in arbitrary units. Data depicted as geometric mean with 95% confidence interval. Differences between groups were analyzed using the Mann-Whitney U test, *P < 0.05, ** P<0.01, ***P<0.001. (DOCX) [file ppat.1007500.s001.docx]

**Fig S1.**

**
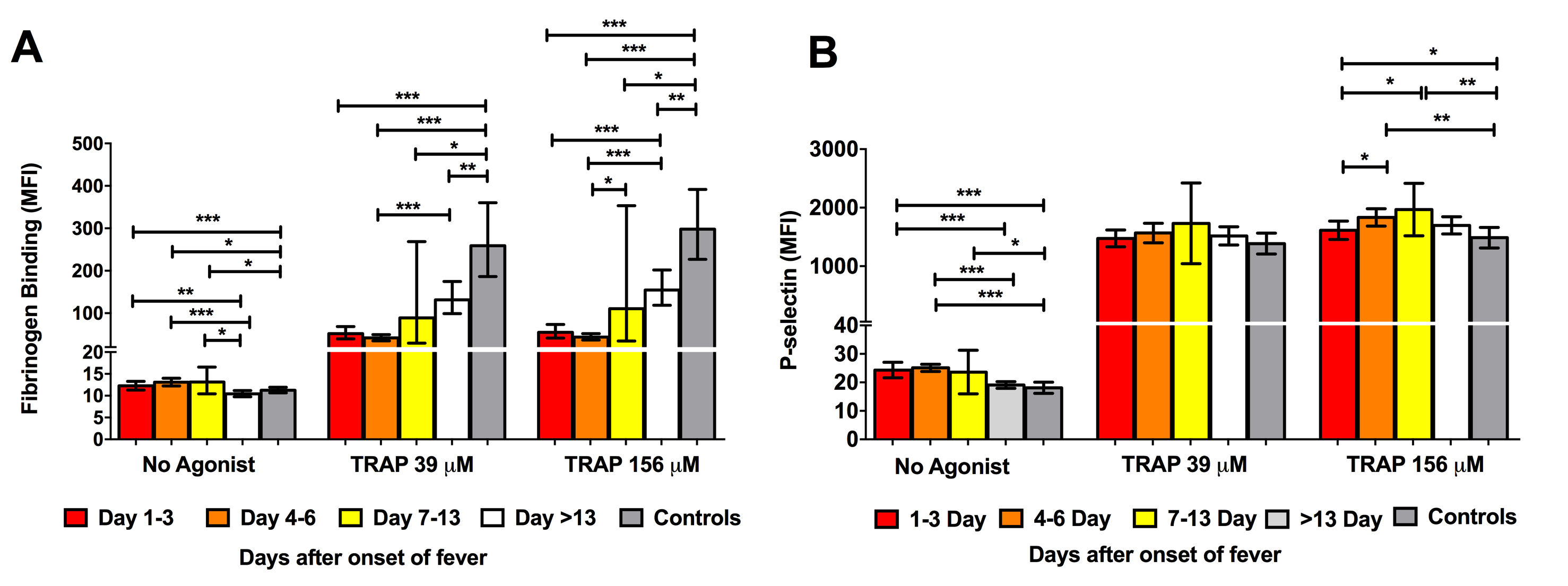
**

**Fig S1.** **Platelet activation with impaired reactivity to TRAP in patients with acute dengue.** Binding of fibrinogen to platelets and platelet P-selectin expression in unstimulated samples and after *ex vivo* stimulation with two concentrations of TRAP. **(A, B)** Data from Bandung cohort with longitudinal data from the different days of fever in dengue patients and in healthy controls. Platelet P-selectin expression and binding of fibrinogen were measured using flow cytometry and are expressed as median fluorescence intensity (MFI) in arbitrary units. Data depicted as geometric mean with 95% confidence interval. Differences between groups were analyzed using the Mann-Whitney U test, **P* < 0.05, ** *P*<0.01, ****P*<0.001.
