# Supplementary material for: Desialylation of platelets induced by Von Willebrand Factor is a novel mechanism of platelet clearance in dengue
Source: PLoS Pathog. 2019 Mar 8;15(3):e1007500. doi: 10.1371/journal.ppat.1007500 (PMC6426266; doi:10.1371/journal.ppat.1007500)
Supplement: S2 Fig — Platelets were gated based on forward and side scatter characteristics (A), followed by positivity for the platelet marker CD61-PC7 (B). The Median fluorescence intensity (MFI) of anti-VWF after stimulation (C) without agonist and (D) after ex vivo VWF-activation with ristocetin (0.777 μM). (E) Observed differences in VWF binding to platelet with marker Anti-VWF-FITC in unstimulated sample and after ex vivo stimulation of 0.777 μM. (DOCX) [file ppat.1007500.s002.docx]

**Fig S2.**

| \| **A** \| **B Based on Gate P0** \| \| --- \| --- \| \| 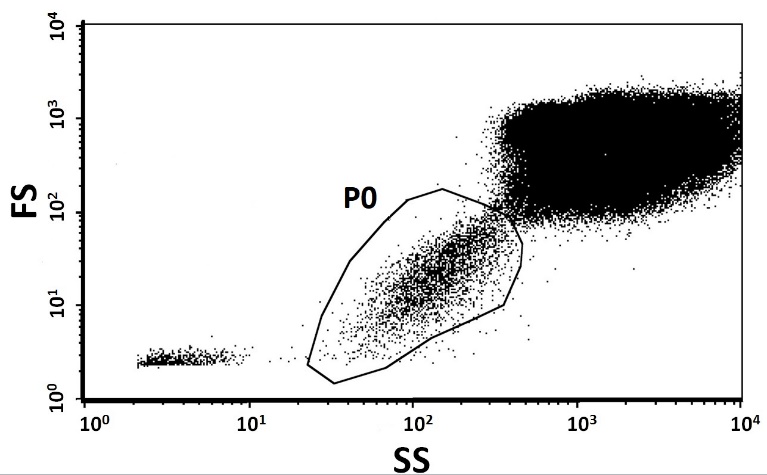 \| 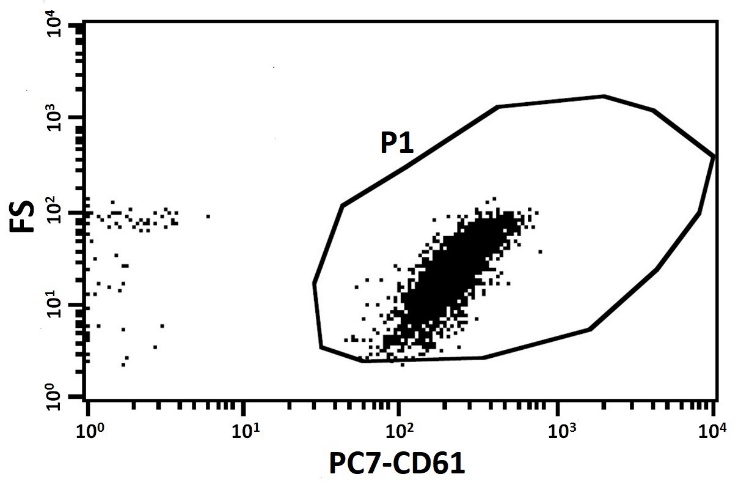 \| \| **C Based on Gate P1** \| **D Based on Gate P1** \| \| 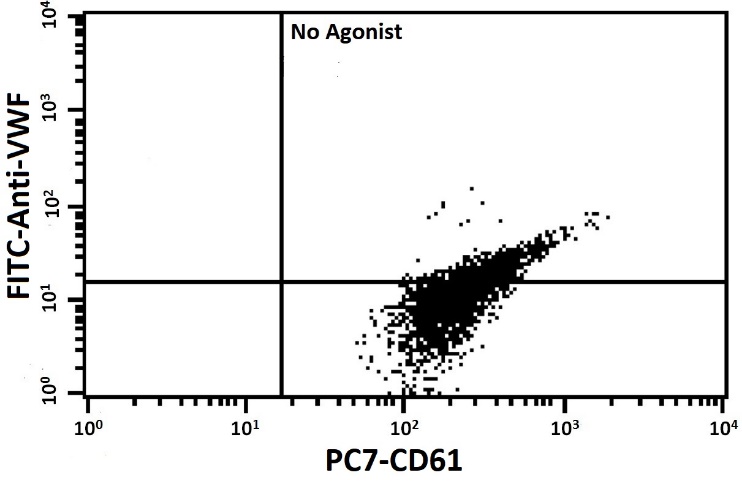 \| 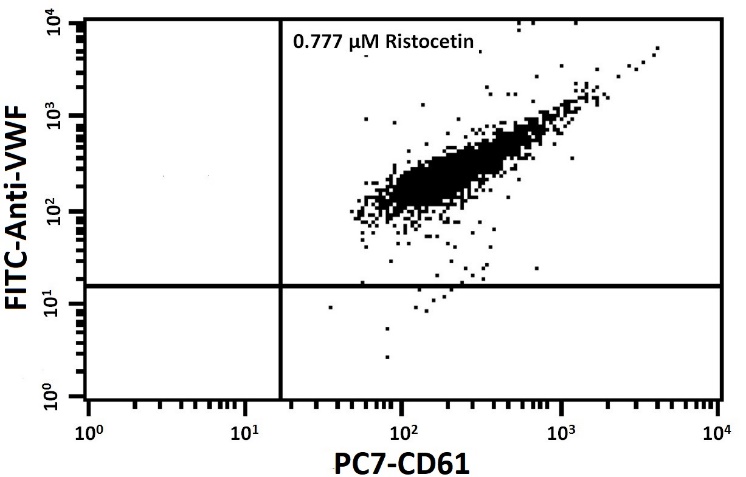 \| \| **E Based on Gate P1** \|  \| \| 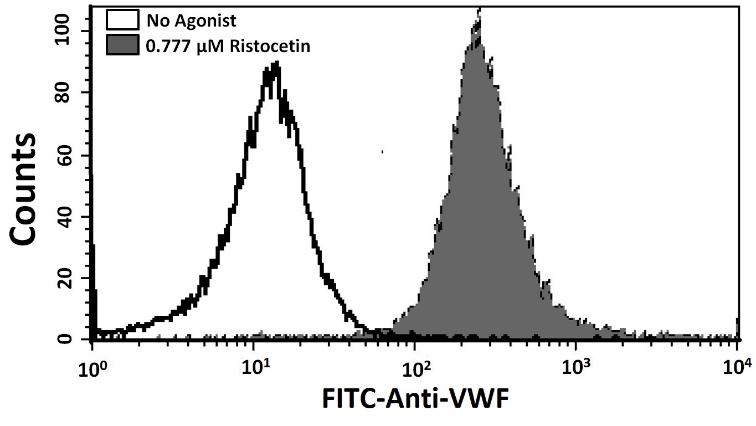 \|  \| |  |
| --- | --- | --- | --- | --- | --- | --- | --- | --- | --- | --- | --- | --- | --- |

**Fig S2. Flow cytometry gating strategy for determination of VWF binding to platelets.** Platelets were gated based on forward and side scatter characteristics **(A)**, followed by positivity for the platelet marker CD61-PC7 **(B)**. The Median fluorescence intensity (MFI) of anti-VWF after stimulation **(C)** without agonist and **(D)** after *ex vivo* VWF-activation with ristocetin (0.777 µM). **(E)** Observed differences in VWF binding to platelet with marker Anti-VWF-FITC in unstimulated sample and after *ex vivo* stimulation of 0.777 µM.

analyzed using the Mann-Whitney U test, **P* < 0.05, ** *P*<0.01, ****P*<0.001.
