# Supplementary material for: Desialylation of platelets induced by Von Willebrand Factor is a novel mechanism of platelet clearance in dengue
Source: PLoS Pathog. 2019 Mar 8;15(3):e1007500. doi: 10.1371/journal.ppat.1007500 (PMC6426266; doi:10.1371/journal.ppat.1007500)
Supplement: S3 Fig — (A) Data from different days of fever in dengue patients and in healthy controls. Data are shown as geometric mean with 95% confidence interval. Differences between groups were analyzed using the Mann-Whitney U test. (B-D) The correlation between VWF binding to platelets without any agonist stimulation and plasma VWF, VWF activation factor and ADAMTS13 activity is shown. Analysis were done using Pearson correlation coefficient. *P < 0.05, ** P<0.01, ***P<0.001, **** P<0.0001. (DOCX) [file ppat.1007500.s003.docx]

**Fig S3.**

**
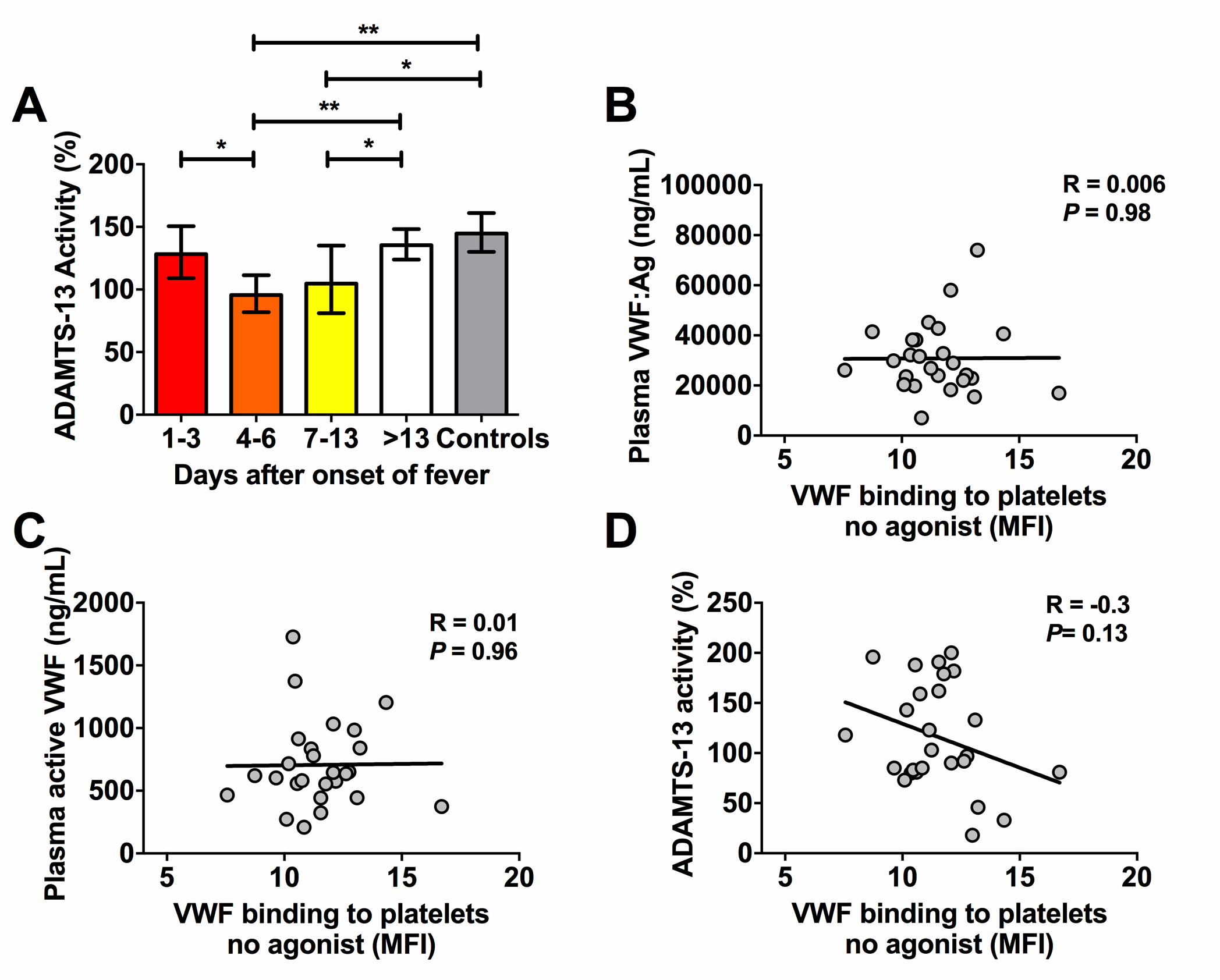
**

**Fig S3. ADAMTS-13 activity in Bandung cohort**. (**A**) Data from different days of fever in dengue patients and in healthy controls. Data are shown as geometric mean with 95% confidence interval. Differences between groups were analyzed using the Mann-Whitney U test. (**B-D**) The correlation between VWF binding to platelets without any agonist stimulation and plasma VWF, VWF activation factor and ADAMTS13 activity is shown. Analysis were done using Pearson correlation coefficient. **P* < 0.05, ** *P*<0.01, ****P*<0.001, **** *P*<0.0001.
