# Supplementary material for: Desialylation of platelets induced by Von Willebrand Factor is a novel mechanism of platelet clearance in dengue
Source: PLoS Pathog. 2019 Mar 8;15(3):e1007500. doi: 10.1371/journal.ppat.1007500 (PMC6426266; doi:10.1371/journal.ppat.1007500)
Supplement: S4 Fig — Data shown are platelet numbers (A and B), VWF binding to platelets in the absence of an agonist (MFI) (C and D), Plasma VWF:Ag levels (E and F) and plasma active VWF levels (G and H). Differences between groups were analyzed using the Mann-Whitney U test, *P < 0.05, ** P<0.01, ***P<0.001. (DOCX) [file ppat.1007500.s004.docx]

**Fig S4.**

**
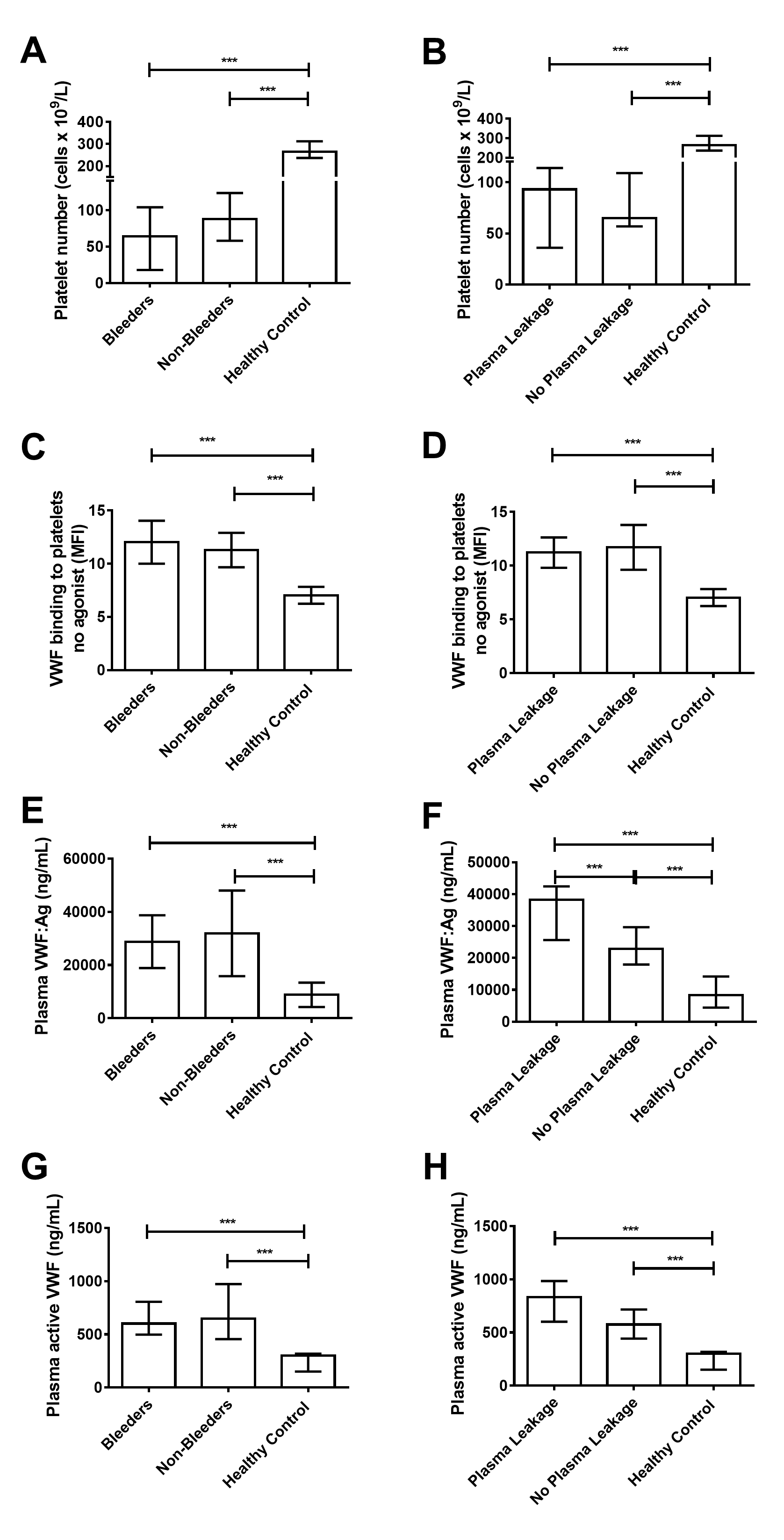
**

**Fig S4. Differences in platelets and VWF parameters between dengue patients with and without bleeding, and patients with and without plasma leakage.** Data shown are platelet numbers (**A** and **B**), VWF binding to platelets in the absence of an agonist (MFI) (**C** and **D**), Plasma VWF:Ag levels (**E** and **F**) and plasma active VWF levels (**G** and **H**). Differences between groups were analyzed using the Mann-Whitney U test, **P* < 0.05, ** *P*<0.01, ****P*<0.001.
