# Supplementary material for: Desialylation of platelets induced by Von Willebrand Factor is a novel mechanism of platelet clearance in dengue
Source: PLoS Pathog. 2019 Mar 8;15(3):e1007500. doi: 10.1371/journal.ppat.1007500 (PMC6426266; doi:10.1371/journal.ppat.1007500)
Supplement: S5 Fig — Platelets were gated in P0 based on forward and side scatter characteristics (A) followed by positivity of the platelet marker CD61-PC7 in P1 (B). The median fluorescence intensity (MFI) of PE-labeled SNA lectin on platelets is determined from gate P1 (C, higher expression, and D, lower expression of sialic acid). (DOCX) [file ppat.1007500.s005.docx]

**Fig S5.**


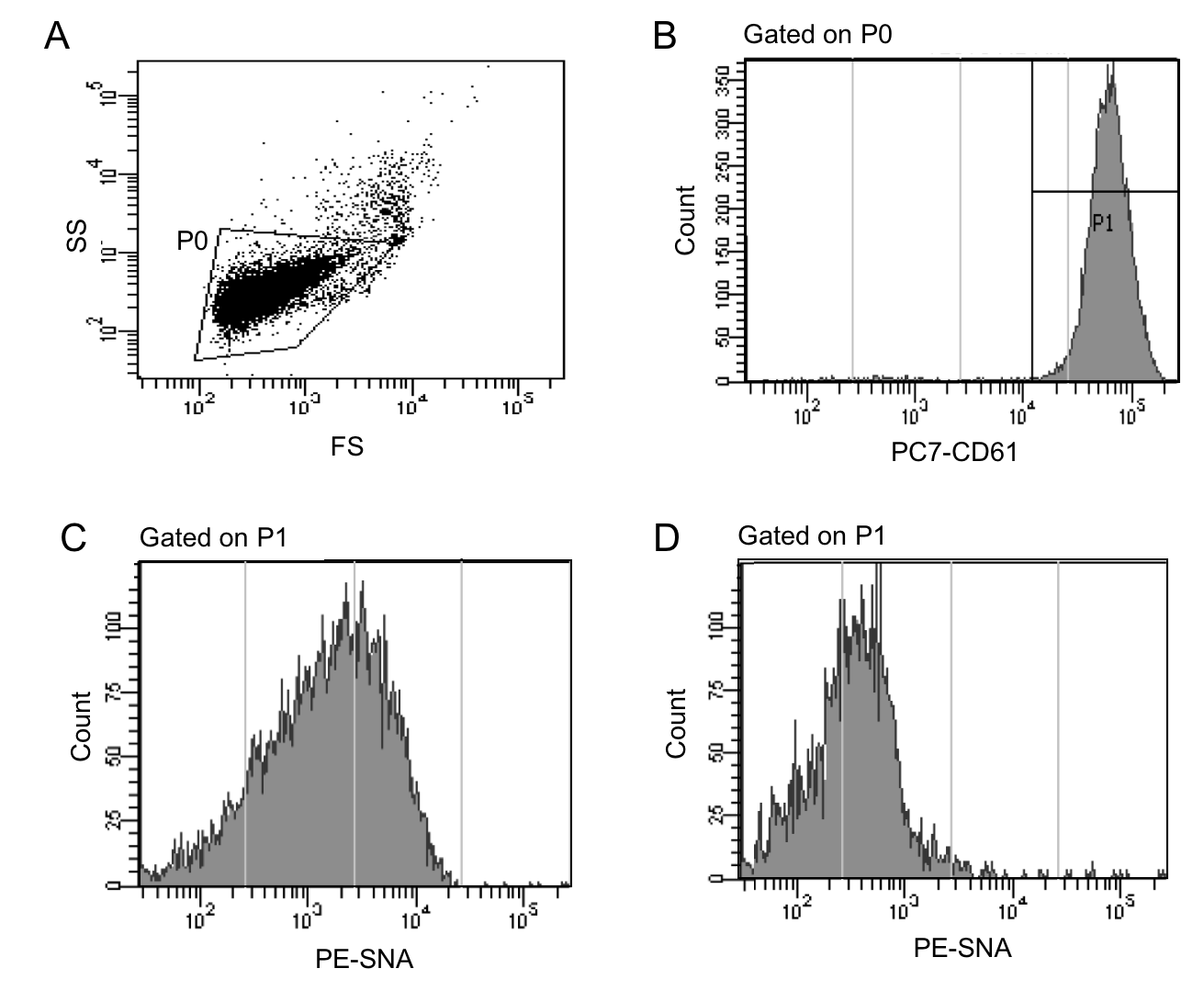


**Fig S5. Flow cytometry gating strategy for determination of sialic acid expression on platelets.** Platelets were gated in P0 based on forward and side scatter characteristics (**A**) followed by positivity of the platelet marker CD61-PC7 in P1 (**B**). The median fluorescence intensity (MFI) of PE-labeled SNA lectin on platelets is determined from gate P1 (**C**, higher expression, and **D**, lower expression of sialic acid).
