# Supplementary material for: Desialylation of platelets induced by Von Willebrand Factor is a novel mechanism of platelet clearance in dengue
Source: PLoS Pathog. 2019 Mar 8;15(3):e1007500. doi: 10.1371/journal.ppat.1007500 (PMC6426266; doi:10.1371/journal.ppat.1007500)
Supplement: S6 Fig — Binding of the lectins (A) SNA and (B) MAL-II to platelet sialic acid residues were measured by flow cytometry in dengue patients with (n = 22) and without bleeding (n = 18). Platelet P-selectin expression and binding of fibrinogen to platelets in unstimulated samples and after ex vivo stimulation with two concentrations of ADP (C, D). Platelet P-selectin expression and binding of fibrinogen were measured using flow cytometry and are expressed as median fluorescence intensity (MFI) in arbitrary units. Data are expressed as geometric mean with 95% CI. Differences between groups were analyzed using the Mann-Whitney U test, *P < 0.05, ** P<0.01, ***P<0.001. (DOCX) [file ppat.1007500.s006.docx]

**Fig S6.**

**Fig S6. Sialic acid expression and platelet reactivity in dengue patients with or without bleeding.** Binding of the lectins (**A**) SNA and (**B**) MAL-II to platelet sialic acid residues were measured by flow cytometry in dengue patients with (n=22) and without bleeding (n=18). Platelet P-selectin expression and binding of fibrinogen to platelets in unstimulated samples and after *ex vivo* stimulation with two concentrations of ADP (**C**, **D**). Platelet P-selectin expression and binding of fibrinogen were measured using flow cytometry and are expressed as median fluorescence intensity (MFI) in arbitrary units. Data are expressed as geometric mean with 95% CI. Differences between groups were analyzed using the Mann-Whitney U test, **P* < 0.05, ** *P*<0.01, ****P*<0.001.
