# Supplementary material for: Desialylation of platelets induced by Von Willebrand Factor is a novel mechanism of platelet clearance in dengue
Source: PLoS Pathog. 2019 Mar 8;15(3):e1007500. doi: 10.1371/journal.ppat.1007500 (PMC6426266; doi:10.1371/journal.ppat.1007500)
Supplement: S7 Fig — (A) Expression of Neuraminidase 1 (Neu-1) and binding of RCA lectin and VWF to platelets after incubation with two concentrations of DENV NS1 protein for 4 hrs at 37°C (n = 7 platelet donors). (B) Binding of VWF and RCA to platelets after incubating washed platelets with increasing concentrations of purified VWF and 1.6mg/ml of ristocetin for 1 hr at 37°C (n = 5 platelet donors). Purified neuraminidase from C. perfringens (100 mU) was used as positive control. Samples were analyzed using Beckman coulter Cytoflex flow cytometry. Data are shown as geometric mean with 95% confidence interval. Differences between groups were analyzed using the Mann-Whitney U test, *P < 0.05, ** P<0.01, ***P<0.001. (DOCX) [file ppat.1007500.s007.docx]

**Fig S7.**

**
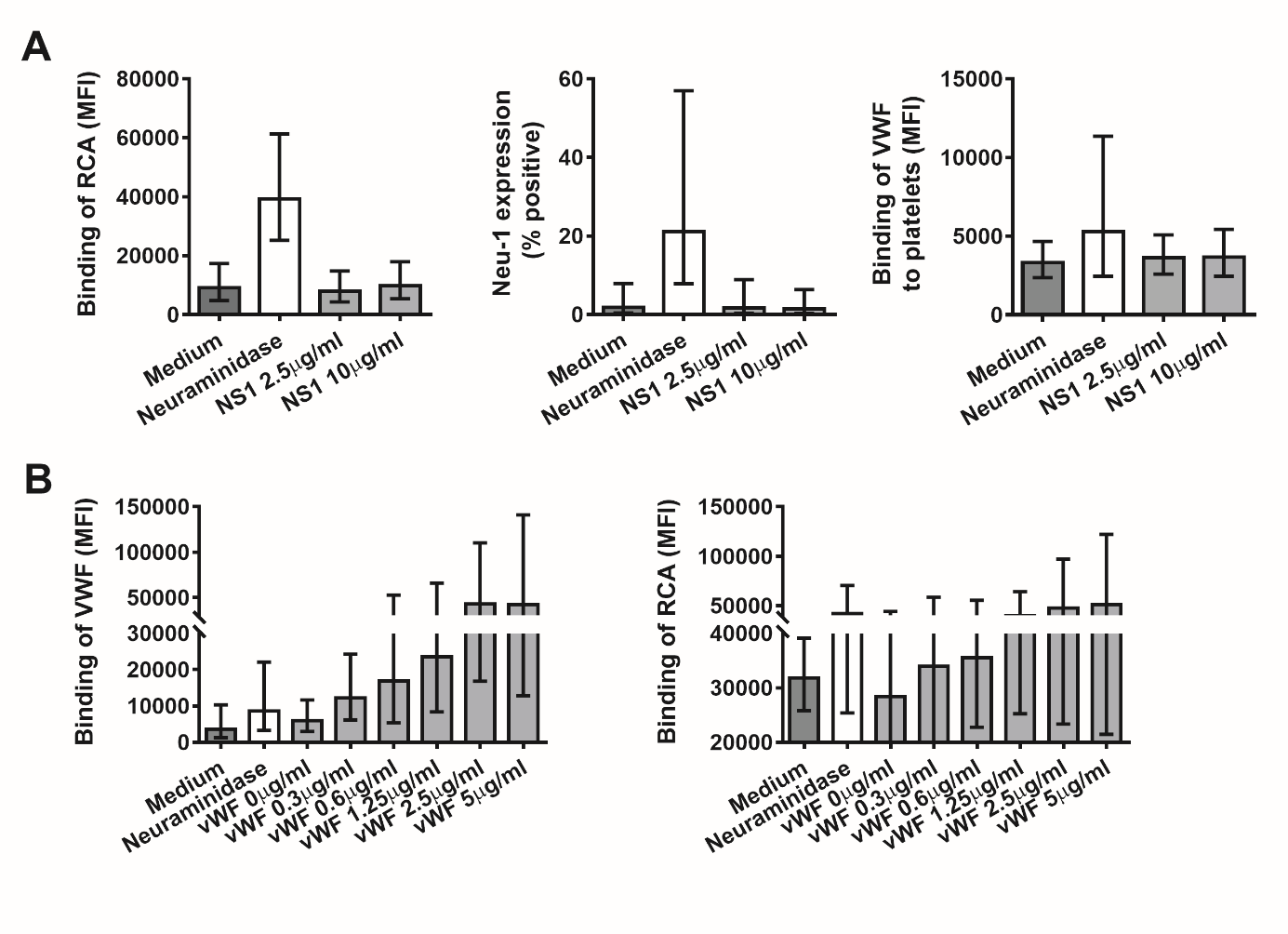
**

**Fig S7. Platelet desialylation is mediated by VWF binding to platelets**. **(A)** Expression of Neuraminidase 1 (Neu-1) and binding of RCA lectin and VWF to platelets after incubation with two concentrations of DENV NS1 protein for 4 hrs at 37°C (n=7 platelet donors). **(B)** Binding of VWF and RCA to platelets after incubating washed platelets with increasing concentrations of purified VWF and 1.6mg/ml of ristocetin for 1 hr at 37°C (n=5 platelet donors). Purified neuraminidase from *C. perfringens* (100 mU) was used as positive control. Samples were analyzed using Beckman coulter Cytoflex flow cytometry. Data are shown as geometric mean with 95% confidence interval. Differences between groups were
